# How a Man Goes to Bed

**Published:** 1880-12

**Authors:** 


### HOW A MAN GOES TO BED.

Speaking of how a man goes to bed, an exchange says :

"There's where a man has the advantage. He can undress in a cold room and have his bed warm before a woman has got her hairpins out and her shoes untied."

That's how it looks in print, and this is how it is in reality : "I am going to bed, my dear. It's half-past ten." No reply. "Now, John, you know you'r always late in the morning. Do get to bed !" "Yes, in a minute," he replies, as he turns the paper wrong side out and begins a lengthy article headed "The Louisiana Muddle." Fifteen minutes later she calls from the bedroom : "John, come to bed, and not keep the gas burning here all night," and murmuring something about "the bill being big enough now," she creeps between the cold sheets, while John sits placidly on, his feet across the piano stool and a cigar in his mouth. By-and-by he rises, yawns, stretches himself, throws the paper on the floor, and seizing the shaker proceeds to that vigorous exercise, shaking the coal stove. Just at this stage a not altogether pleasant voice inquires: "For pity's sake ! ain't you ready for bed yet ?" "Yes, yes, I'm coming ! Why don't you go to sleep and let a fellow alone ?"

Then he discovers that there's coal needed. When that is supplied and rattled into the stove he sits down to warm his feet. Next he slowly begins to undress, and as he stands scratching himself and absently gazing on the last garment, dangling over the back of the chair, he remembers that the clock is not wound yet. When that is attended to he wants a drink of water, and away he promenades to the kitchen. Of course, when he returns his skin resembles that of a picked chicken, and once more he seats himself before the fire for a last "warm-up." As the clock strikes twelve he turns out the gas, and with a flop of the bed-clothes and a few spasmodic shivers he subsides—no, not yet ; he forgot to see if the front door was locked, and another flop of the bed-clothes brings forth the remark : "Good gracious ! if that man ain't enough to try the patience of Job !" Setting her teeth hard she awaits the final flop, with the accompanying blast of cold air, and then quietly inquires "if he is settled for the night," to which he replies by muttering : "If you ain't the provokingest woman !"

